# Gender diversity and the twin relationship: “If I’m feeling this way, shouldn’t my twin be feeling this way too?”

**DOI:** 10.1080/26895269.2025.2552337

**Published:** 2025-11-07

**Authors:** Will Conabere, Louise Bourchier, Anja Ravine, Ken C. Pang, Sue Malta

**Affiliations:** a Murdoch Children’s Research Instituteb‑ Department of Paediatrics, UoMc‑Sexual Health Unit, MSPGHd ‑ Department of Adol Med, RCHe ‑ Twins Research, Australia, Victoria, UoM; b Department of Paediatrics, University of Melbourne, Melbourne, Victoria, Australia; c Sexual Health Unit, Melbourne School of Population and Global Health, University of Melbourne, Melbourne, Victoria, Australia; d Department of Adolescent Medicine, The Royal Children’s Hospital Melbourne, Parkville, Victoria, Australia; e Twins Research Australia, University of Melbourne, Melbourne, Victoria, Australia

**Keywords:** Transgender, Twin relationship, Gender Diversity, Twin studies, Family dynamics

## Abstract

**Background:**

Twin studies have been pivotal in advancing knowledge of genetic influence on gender diversity. However, the extent to which the unique dynamics of the twin relationship may also influence gender formation, and potentially distinguish twins from the broader trans community, remains unknown.

**Method:**

This study recruited 11 young people from the Royal Children’s Hospital Gender Service in Melbourne, Australia, and conducted semi-structured interviews, investigating the interplay between gender diversity and the twin relationship. The interviews were analyzed using Inductive Content Analysis with three coders.

**Results:**

The results found that for most trans participants, their twin relationship either served as a source of support or had no notable impact on their gender journey. Similarly, cisgender participants with a trans co-twin did not report any significant consideration or questioning of their own gender identity prompted by their twin’s coming out. In most cases, the twin relationship was maintained or strengthened after the coming out process, particularly in the long term.

**Conclusion:**

Overall, the twin relationship likely functions similarly to other close relationships (i.e. parents, non-twin siblings), notably the potential to provide affirming support, rather than contributing to a twin-specific experience. However, further research is required to explore these dynamics in greater depth.

## Introduction

Numerous studies have demonstrated the influence of the twin relationship on various aspects of development, including socio-behavioral characteristics (Robin & Casati, 1994; Rutter & Redshaw, 1991), behavioral differences (Moilanen et al., 1999), identity differentiation (Watzlawik, 2009) and personality differences (Pulkkinen et al., 2012). Collectively, these works highlight that for specific traits, twins can influence developmental trajectories compared to the experience of singletons (non-twins). Despite these insights, no research has explored the intersection of the twin relationship in the context of gender diversity, leaving unanswered the question of whether gender identity formation may be influenced by the unique dynamics of twinship.

This gap is particularly notable given that much of the research into the genetic contribution to gender diversity has relied on twin studies (Conabere et al., 2025a; Karamanis et al., 2022; Polderman et al., 2018). These studies, which typically measure concordance (both twins being gender diverse) and discordance (only one twin identifying as gender diverse), downplay the possibility of environmental influences from the twin relationship. However, it is unclear at this point whether twins experience unique gender trajectories compared to singletons. Given that many transgender and gender diverse (trans) individuals report their first awareness of sex-gender dissonance before adolescence (Zaliznyak et al., 2021), ethnographic research focusing on the gendered experiences of twins in their formative years could yield valuable insights on this topic.

Qualitative research on other family dynamics, such as parent-child or sibling relationships have highlighted both supportive and challenging influences arising from close family bonds (Andrzejewski et al., 2021; Parker & Davis-McCabe, 2021). More recent research has also explored experiences of families with more than one trans family member (Riggs et al., 2025). While family relationships often provide essential support and enrichment, destabilizing fears and tensions may emerge in response to a family member having confided their gender diverse identity (Andrzejewski et al., 2021; Parker & Davis-McCabe, 2021; Westwater et al., 2020; Wheeler et al., 2019). Given that twin relationships are often much closer than those of singleton siblings (Koch, 1966; Neyer, 2002; Segal, 1999; Tancredy & Fraley, 2006), it is possible that their gendered experiences may include twin-specific dynamics that remain undefined. The event of one twin confiding their sense of gender to the other could alter the twin dynamic or the co-twin’s own gender-related experiences.

As qualitative data are well suited to explore the ‘how’ and ‘why’ of a particular phenomenon (Sofaer, 1999; Tenny et al., 2023), we hypothesized that qualitative interviews with gender diverse twins may provide additional insights into how twins navigate their identities and relationships, particularly after a significant event such as one twin’s “coming out”. This research addresses a gap in understanding the unique experiences of gender diverse twins, offering valuable insights on family dynamics, and complementing and contextualizing quantitative twin research on gender diversity. Thus, the project aimed to explore these dynamics of gender diversity and twinship within a small Australian clinical sample of young people.

## Materials and methods

### Participants and recruitment

Participants included transgender, non-binary, and other gender diverse (hereafter, trans) adolescents and young adults (under 21 years of age) recruited through the Royal Children’s Hospital Gender Service (RCHGS), a specialized pediatric gender service in Victoria, Australia. Systematic clinical data collection at RCHGS includes data on whether the young people attending the service are a twin, which was used to identify 59 individuals eligible for the present study. Eligible families (including the young person identified systematically and their co-twin) were first contacted by the clinical service, which informed them about the study and offered the option to decline contact from the research team. The remaining families were contacted *via* email, with non-respondents receiving a follow-up SMS at 4 wk and a follow-up phone call at 8 wk. Eligible families were informed that declining to participate would not affect their clinical care at RCHGS and that no funds were available for remuneration. As the RCHGS supports individuals under the age of 18, most invitations went directly to parents/guardians. When parents responded or shared details of the study with their children, parental consent was considered implicit and written consent was not sought. The participant provided informed consent, independently or alongside their parent, by completing the online REDCap consent form prior to the interview. While both twins in each pair were encouraged to participate, individuals from pairs where only one twin participated were still included in the project. Eleven individuals (5 twin pairs and 1 individual twin) were interviewed.

### Measures

Interviews were conducted online using Zoom, which included audio-recording. A semi-structured interview guide of questions of interest was developed (Supplementary Material 1), which allowed both the researcher and participants to raise related matters, if they seemed potentially relevant to the theme of gender identity (DeJonckheere & Vaughn, 2019). These questions covered topics such as personal identity, the twin relationship across different time points, and the participants’ gender journey, both in general and in the context of their relationship with their twin. Consent for an audio-recording was sought before each interview; with all participants providing consent. Audio recordings were subsequently electronically transcribed (Archibald et al., 2019). Prior to analysis transcripts were manually checked twice for errors or misspellings.

Interviews ranging from 15 to 38 min duration were evaluated using Inductive Content Analysis, which identifies classifications that emerge from the data itself, rather than those predetermined by the researchers or from the literature (Erlingsson & Brysiewicz, 2017; Forman & Damschroder, 2007; Hsieh & Shannon, 2005; Vears & Gillam, 2022). This analysis method categorized and organized participants’ views and experiences with quotes taken at face-value and no attempt at deeper abstraction or interpretation. The method was selected due to the absence of literature providing guidance on anticipated themes that may be encountered, particularly across different interviews (DeJonckheere & Vaughn, 2019). This approach also aligned with the project’s aim to explore how gender diverse twins navigate their identities and relationships, allowing participants’ experiences to be presented clearly without imposing interpretive frameworks.

To achieve familiarity with the data, all transcripts were firstly read multiple times by the primary coder (WC). The transcripts were then analyzed according to the method of Erlingsson and Brysiewicz (2017). To achieve this, the primary coder divided transcripts into textual “meaning units”, consisting of short direct quotes from participants, which were then used for analysis. Meaning units were summarized and contextualized to give rise to “condensed meaning units”, which were then assigned a “code”, a short description of the content of the condensed meaning unit, which was no longer than a few words.

All meaning units, condensed meaning units and codes were then copied into an Excel spreadsheet. Related codes were grouped together to create “categories”, and categories grouped to create overarching “topics”. Formation of categories and topics involved comparison of the 11 interviews to ensure categories and topics present in multiple interviews were assigned the same term. The second coder (LB) then read 5 of the transcripts and created a data-map of categories and topics. The third coder (SM) completed the same process as Coder 2 with 3 transcripts. In this manner, a process of coding validation was conducted, optimizing the analysis.

Due to the comparative rarity of being both a twin and gender diverse, it is possible that some individuals could be inadvertently identified. To preserve the anonymity of participants all quotes are deidentified, and ages are provided as a range (i.e. 7–11, 12–16, 17–21 years). Further, while all quotes are presented verbatim as they occurred in the interviews, it has been necessary at times to assist with clarity by adding in words. These are represented in square brackets to distinguish them from the original text. Further, all names have been replaced with “Participant Number(s)” and all other identifying references have also been removed. Both to honor and maintain the authenticity of responses, care was taken to ensure no misquoting or inferences were made.

### Reflexivity

We acknowledge that the positionality of the research team is an important consideration when interpreting our study findings. Our team includes members affiliated with a pediatric gender-affirming specialist clinic for trans young people in Victoria, Australia, and brings diverse research and professional experience, particularly in gender and sexual health. We also hold a range of personal identities and experiences, including gay, straight, cis, and trans team members. This investigation is grounded in our shared commitment to understanding and improving the experiences of trans young people.

### Ethics, recruitment and consent

The Royal Children’s Hospital Human Research Ethics Committee granted approval for the study (#91711). Consent was obtained for all participants, including parental consent for legal minors (younger than 18 years).

## Results

The 11 participants represented four monozygotic (identical) twins, one complete dizygotic (non-identical) pair and one individual from a dizygotic pair. Participants comprised transgender males (*n* = 2), transgender females (*n* = 2), non-binary individuals (*n* = 3) and cisgender siblings (*n* = 4). All participants were Caucasian; further demographic details for each individual/pair are presented in Table 1.

**Table 1.** Demographics of participant pairs (gender identity, age, zygosity, concordance).

Participant number	Gender identity	Age group (years)	Twin Zygosity	Concordance
1A	Non-binary person	17–21	Identical (MZ)	Concordant
1B	Transgender male			
2A	Non-binary person	7–11	Identical (MZ)	Discordant
2B	Cisgender female			
3A	Cisgender female	12–16	Identical (MZ)	Discordant
3B	Transgender male			
4A	Transgender female	12–16	Non-identical (DZ)	Discordant
4B	Cisgender female			
5A	Non-binary person	17–21	Non-identical (DZ)	Discordant
6A	Transgender female	7–11	Identical (MZ)	Discordant
6B	Cisgender male			

### Demographics

#### Findings

Two topics were identified from the Inductive Content Analysis, including (i) the potential influence of the twin relationship on gender identity formation; (ii) ensuing influences on the twin relationship. Within these two topics, there were multiple categories outlined in Table 2, which are presented in decreasing order of how common they were within the data (most common listed first).

**Table 2.** Inductive content analysis topics and categories.

Topic	Categories
**1.** Influence of twin relationship on gender discovery (own or twin’s) and coming out process.	Twin relationship helped/supported gender self-recognition Twin relationship did not influence gender self-recognition Twin relationship hindered gender self-recognition/coming out process Influence of co-twin’s coming out on other twin’s sense of gender
**2.** Influence of gender diversity (own or twin’s) on twin relationship.	Gender diversity did not impact the twin relationship Gender diversity and strength of the twin relationship Gender diversity disrupted the twin relationship

##### Topic 1: Influence of the twin relationship on gender discovery and coming out process

This section describes the impact that the participant’s co-twin had on their self-awareness of gender and the coming out process. The three major common response elements included (i) support from their twin, (ii) process uninfluenced by the twin dynamic, (iii) process hindered by the twin relationship. A less prominent response element was (iv) the impact on one’s gender identity from having a gender diverse twin.

###### Category 1: Co-twin supported gender diversity

A major theme was reassuring support provided by the co-twin during the coming out process. In some of these instances, this support was directly attributed to being a twin.

**Participant 3B:** “*I think it’s because [Participant 3A] is my twin…It’s such a close bond, that it made it a lot easier*.”

**Participant 5A:** “*So yeah, I think [they’ve] been [a] very big part of my journey into feeling comfortable talking about it and feeling comfortable expressing who I am*.”

For some, the anticipated support from their co-twin was also a key factor in facilitating their coming out process. Despite not being fully aware of how their twin would initially react, the expectation of being accepted by their co-twin was helpful.

**Participant 5A:** “*But at the same time, like I knew [they] would be nothing but supportive towards me, which I’m very, very lucky I could have that experience*.”

**Participant 1A:** “*I think it was easier because I knew immediately [that] I was going to be accepted*.”

**Participant 1B:** “*Probably because I knew out of anyone that [they’d] be chill with it. Like, I don’t know, I just knew what [they] would be like, I already knew how [they] would react to it—so it wasn’t really scary*."

For the concordant pair specifically, when the second twin was ready to reveal their gender identity, they implicitly knew that their twin would understand. Their twin’s knowledge of gender diversity and related dysphoria allowed them to feel comfortable when they were ready to share their gender identity.

**Participant 1A:** “*I knew since he had been dealing with gender, like he would understand what I meant if I just went ‘use they/them’*.”

Within a birth-registered sex discordant pair (i.e. male-female), the gender diverse twin expressed benefit from visualizing themself as their twin’s gender. Observing their twin’s physical changes during adolescence also allowed this participant to understand the changes they wished for themselves.

**Participant 4A:** “*Oh when I was younger, I think I felt like, I don’t know she looked a bit different to me and I don’t know, but I felt the same as her kind of*.”

###### Category 2: No influence from the twin relationship

While participants emphasized that the twin relationship specifically was beneficial in coming out, many felt that it did not influence their gendered self-recognition. More specifically, they felt that the timing of their self-recognition occurred independently of being a twin.

**Participant 3B:** “*…but I don’t think it had any impact on the timing*.”

**Participant 1B:** “*I don’t think it made a difference… no. I kind of always knew*”

When asked whether the timing of coming out may have changed if they were not a twin, gender diverse participants did not think this was the case.

**Participant 4A:** “*I don’t really think so*.”

**Participant 6A:** “*I would just tell [mum and dad]* w*hen I did*.”

###### Category 3: Twin relationship hindered or slowed down gendered self-recognition and/or coming out

Some participants felt that the experience of being a twin, or the personal attributes of their co-twin, may have delayed their gendered progression of either self-recognition or disclosing their identity to others.

Among two pairs of discordant MZ twins, initiating conversations about gender diversity led to the their co-twin’s realization that their experience of gender was different, leaving them feeling confused.

**Participant 3B:** “*I did have a couple of thoughts when I was about to come out as in… you know… like if I’m feeling this way, shouldn’t [Participant 3A] be feeling this way as well?*”

**Participant 6A:** “*Kind of because… like we’re supposed to be the same, kind of… since we’re identical twins*.”

The concordant MZ pair had a different experience. The second twin to come out as gender diverse did so several years later. This participant attributed the delay to their own focus, and their family’s focus, on their co-twin’s gender identity during their upbringing.

**Participant 1A:** “*I was focused on [Participant 1B’s] gender, so I didn’t really think about my gender until I had fully worked things out and had time to think about it*” *… “cause I think with [Participant 1B] being transgender, a lot of gender was kind of focused on him*”

When Participant 1B was interviewed, they also acknowledged the distinct focus on their gender identity as a child. Both twins acknowledged that this may have affected Participant 1A’s gender-related timeline.

**Participant 1B:** “*I think it might have made it harder [for them].”… “They didn’t come out until… they left like school and stuff and also they didn’t tell us like any of that sort of stuff for a while until [they had socially transitioned]*”

Participant 1 A also indicated that being a twin still may be impacting their expression of their own gender identity but did not go into further detail about how.

**Participant 1A:** *“… Still hinder[s] it just a little, but probably not as much”*

Other gender diverse participants suggested that being a twin may have contributed to delaying the expression of their gender identity during their younger years. These included the prospect of differing from their twin when they were meant to similar, or concern about how their co-twin would perceive them.

**Participant 5A:** *“I never really wanted to rock the boat too much. In terms of, [they] would see me differently and then I would be a completely different person to [them]”*

**Participant 4A:** *"I think it might have slightly slowed it down, because I don’t know, I was just nervous about what she would think a bit."*

When asked about the extent of the delay arising from these concerns, one participant declared that it only slowed them down a very short amount of time.

**Participant 4A:** *“I don’t know, like a couple of weeks”*

###### Category 4: Influence of co-twin’s coming out on own sense of gender

We asked those who were cisgender how their own sense of gender may have been influenced after learning of their twin’s gender diversity. This question elicited two types of responses: (i) it did not cause them to ponder over their gender, or (ii) they quickly considered their gender identity and confidently discerned that they were cisgender.

**Participant 6B:** *“Not really crossed my mind….”*

**Participant 4B:** *“No, never.”*

**Participant 3A:** *“I mean honestly like… I never really like had a huge like think about it, I guess* … *So, it was just… a very quick thing. I was like, yeah, I’m a girl”*

Trans participants within a discordant pair were asked whether they had discussed gender identity with their twin. Those who had these conversations noted that their twin had never expressed similar feelings, while those who hadn’t were confident that their twin would have shared such feelings if they existed.

**Participant 6A:** *“He usually would just say ‘never’”*

**Participant 5A:** *“No, not really. I’d figured, you know, [they’d] just tell me if [they were] feeling like that or something.”*

Overall, there was no evidence to suggest that gender diversity in one twin induced identity shifts or changes in their co-twin.

##### Topic 2: Influence of gender diversity on the twin relationship

In this section, we discuss the impact that gender (either the participant’s or their co-twin’s) had on the twin relationship more broadly. The two predominant themes to emerge were (i) absence of any perceived change and (ii) strengthened or improved relationships. A third minor topic was raised by a small number of participants, who indicated that (iii) a new less harmonious dynamic had emerged after knowledge of the gender diversity had been shared.

###### Category 1: Unchanged twin relationship

One of the major topics identified was the unchanged nature of the twin relationship after one twin learned about the other’s gender diversity. In particular, trans participants who initially felt anxious about coming out to their co-twin noted that their fears of a changed relationship did not materialize.

**Participant 4A:** *“No, I don’t think she really cared, honestly. I think she just said, ‘oh well’. And just like, went on with her life, I guess.”*

**Participant 3B:** *“And then nothing, yeah, nothing’s really changed with us.”*

**Participant 1B:** *“I remember mentioning it to [them] and I was like, hey, I’m not a girl and [they were] pretty chill about it.”*

Among cisgender co-twins, the most common response was that their closeness or support toward one another remained unchanged.

**Participant 4B:** *“Not really. It wasn’t really something I fully understood, and I didn’t particularly care. It was just like, [Participant 4A] was just [Participant 4A], and I didn’t really care that much.”*

**Participant 3A:** *“I don’t think it did, no. Yeah, I didn’t really feel like it was like all new and different. When he came out - it kind of just like felt quite normal to me, I guess.”*

The concordant twin pair also felt that nothing changed in their relationship when the first twin shared their gender identity with the other.

**Participant 1A:** *“Um It sounds weird when I say it, but I didn’t really care because I was like, oh well – still my twin.”*

**Participant 1B:** *“I remember mentioning it to them and I was like, ‘Hey, I’m not a girl’ and they were pretty chill about it. Cause I didn’t say that I was trans or anything. I just said that I wasn’t a girl.”*

One other participant explained that although supportive of their twin’s gender diversity, they remained challenged by the associated changes in gendered forms of expression.

**Participant 4B:** *“that’s still kind of the same just ‘cause…[it] hasn’t been that long, and it’s just something I can try to still get used to after like 12 years of something else.”*

It is clear from these responses that the majority of participants reported no lasting negative impact on their relationships. The second most frequently expressed sentiment was positive change, outlined below.

###### Category 2: Strength of the twin relationship

The other major impact identified was a strengthened twin relationship after participants shared their gender diversity with their twin. Many trans participants felt supported by their co-twin immediately following this disclosure.

**Participant 3B:** *“Again, everyone’s been really supportive, and [Participant 3A] did help me come out to my friends and stuff.”*

Participants also acknowledged that they knew their twins would be supportive prior to informing them of their gender identity. When prompted, they described their twin relationship as remaining strong.

**Participant 1B:** *“I knew she was going to be supportive”*

**Participant 5A:** *“I knew [they] would be nothing but supportive towards me, which I’m very, very lucky I could have that experience.”*

###### Category 3: Gender diversity challenged the strength of the twin relationship

There were a small number of participants who explained that their co-twin’s new gender identity or gender expression gave rise to new stresses in their twin relationship. For one pair, some disagreements emerged.

**Participant 4B:** *“A little bit, it’s changed like a bit of stuff. And there’s been like some arguments over it.”*

However, once prompted to elaborate on these discussions, the participant described common sibling arguments and dynamics, such as sharing belongings.

Another pair acknowledged that they had drifted apart since coming out. Notably, both instances of relationship strain were observed in the younger participants.

**Participant 6B:** *“We’re drifting off from each other, like getting less close.”*

**Participant 6A:** *“It changed when I got a little bit older, and then he started to just be mean and tease.”*

However, the twins also acknowledged that this was coincident with the phase of coming out, rather than being the cause. The participants noted that it may have been due to their different interests and sibling rivalry, rather than specifically related to the topic of gender.

**Participant 6A:** *“Because I find that since I’m a girl, [Participant 6B] is finding more ways to annoy me.”…. “I, like- I’m just different. I sort of like different stuff, but I still kind of am interested into stuff that I used to.”*

The question of whether a twin pair could still be considered “identical” after one had transitioned, was raised by one participant. While on some level they were aware that as twins they would always be genetically identical, the thought was of some concern at the time.

**Participant 1A:** *“my main concern when he came out with his gender was, I was like, wait, does that mean we’re still identical twins”.*

Responses overall indicated that the twin relationships more often served as a source of support for trans participants.

## Discussion

This exploratory study provides preliminary insights into the intersection of gender diversity and the twin relationship, specifically among young people. Findings suggest that despite the often distinct and uniquely close nature of twin relationships, they did not seem to alter the participants’ experiences of gender, nor their coming out process. Further, there was no description of a trans co-twin’s disclosure evoking a flow-on altered awareness of gender diversity in the other. Instead, the twin relationship predominantly served as a source of emotional support or remained a neutral aspect of their experiences. This aligns with previous research on the supportive roles of family members and friends for gender diverse individuals (Andrzejewski et al., 2021; Parker & Davis-McCabe, 2021; Wheeler et al., 2019), suggesting that the twin relationship may function similarly to other close relationships (i.e. parents, non-twin siblings) of empathic support, which often becomes affirming.

While many findings aligned with previously reported qualitative studies on siblings and families (Andrzejewski et al., 2021; Parker & Davis-McCabe, 2021; Westwater et al., 2020; Wheeler et al., 2019), some experiences were unique to twins. For example, gender-diverse participants with cisgender co-twins described initial confusion upon realizing that their typically similar experiences did not align as they had come to expect. Among discordant pairs, a few participants suggested that being a twin may have delayed their coming out process, though most struggled to articulate why. A delay was also noted in the sole concordant pair interviewed, where the second twin’s coming out process seemed delayed rather than accelerated by the earlier emergence of their co-twin’s trans identity. However, the participant from this pair explained that this delay would likely have occurred with any sibling, twin or not, and was not specific to their experience as a twin. Since only one concordant pair was included in the study, these findings provide limited insight into the diverse experiences of concordance in twins. Future research with larger samples or a specific focus on concordance is needed to explore further these dynamics.

Shifting focus to the inverse dynamic—the impact of an individual’s gender affirmation journey on the twin relationship—most participants reported that coming out to their twin did not significantly alter their bond. Interestingly, in our sample, younger participants noted a slight drifting in their relationship, while older participants described periods of distance followed by a strengthening of their long-term closeness. These observations suggest a potential influence of age and maturity on research findings in this area. Following up with the younger twins as they grow older could provide deeper insights into how these dynamics evolve.

Twin pairs where one or both are gender diverse, although a niche population, are critical to twin studies investigating the origins of gender diversity (i.e. the relative contributions of genes as well as shared and non-shared environmental exposures) (Conabere et al., 2025b; Polderman et al., 2018). This study aimed to explore whether the twin dynamic may impart an additional environmental compartment requiring consideration in twin-based research focussing on gender identity. However, our findings do not suggest that this is the case. The dynamics observed in this study were similar to those seen in research on parent-child and sibling relationships. The participants either reported no reflection on their gender identity, or described brief moments of consideration that did not prompt self-questioning or change.

This exploratory study serves to guide further understanding of the impact of twin relationships on gender diverse individuals. Consequently, the experiences presented in this paper are not exhaustive, particularly for concordant pairs, as only one concordant pair participated. Additionally, the sample was drawn from a clinical gender service, where the extent of familial support is likely to be overrepresented; this limits the representation of experiences from twins in non-supportive family contexts. While efforts were made to recruit a larger and more racially diverse sample, the rarity of being both transgender and a twin posed a significant recruitment challenge. Despite these limitations, the autoethnographic responses in these interviews have provided valuable preliminary insights. Future studies with larger samples, a focus on concordant twins, or an investigation of long-term changes with adult participants will provide a deeper understanding of potential influences of the twin relationship on gender identity and expression.

## Conclusion

This exploratory study offers preliminary insights into the interplay between gender diversity and the twin relationship. Overall, survey responses revealed nothing uniquely twin-specific within the experience of being a gender diverse twin. Further, there were no responses suggesting that the event of learning of the other twin’s atypical gender identity had induced a change of gender identity. Instead, the twin relationship acted as a point of support or indifference in the coming out process.

## Supplementary Material

Supplemental Material

## References

[CIT0001] Andrzejewski, J., Pampati, S., Steiner, R. J., Boyce, L., & Johns, M. M. (2021). Perspectives of transgender youth on parental support: Qualitative findings from the resilience and transgender youth study. *Health Education & Behavior: The Official Publication of the Society for Public Health Education*, *48*(1), 74–81. 10.1177/1090198120965504 PMC7962554 33106050

[CIT0002] Archibald, M. M., Ambagtsheer, R. C., Casey, M. G., & Lawless, M. (2019). Using zoom videoconferencing for qualitative data collection: perceptions and experiences of researchers and participants. *International Journal of Qualitative Methods*, *18*(1), 1–18. 10.1177/1609406919874596

[CIT0005] Conabere, W., Ravine, A., Malta, S., Bourchier, L., Harte, F., Hopper, J. L., & Pang, K. C. (2025a). Using twin data to examine heritable and intrauterine hormonal influences on transgender and gender diverse identities. *Scientific Reports*, *15*(1), e21680. 10.1038/s41598-025-06265-6 PMC12218214 40594415

[CIT0004] Conabere, W., Bourchier, L., Malta, S., Ravine, A., & Pang, K. C. (2025b). Genetic and environmental contributions to gender diversity: A systematic review of the twin literature. 10.1007/s10519-025-10231-3 PMC12494644 40938572

[CIT0006] DeJonckheere, M., & Vaughn, L. M. (2019). Semistructured interviewing in primary care research: A balance of relationship and rigour. *Family Medicine and Community Health*, *7*(2), e000057. 10.1136/fmch-2018-000057 PMC6910737 32148704

[CIT0007] Erlingsson, C., & Brysiewicz, P. (2017). A hands-on guide to doing content analysis. *African Journal of Emergency Medicine: Revue Africaine de la Medecine D’urgence*, *7*(3), 93–99. 10.1016/j.afjem.2017.08.001 PMC6234169 30456117

[CIT0008] Forman, J., & Damschroder, L. (2007). Qualitative content analysis. In Hesse-Biber, S. N. (Ed.), *Advances in mixed methods research: Theories and applications* (Vol. 11, pp. 39–62). Elsevier. 10.1016/S1479-3709(07)11003-7

[CIT0009] Hsieh, H. F., & Shannon, S. E. (2005). Three approaches to qualitative content analysis. *Qualitative Health Research*, *15*(9), 1277–1288. 10.1177/1049732305276687 16204405

[CIT1781855] Karamanis Georgios. Karalexi Maria. White Richard. Frisell Thomas. Isaksson Johan. Skalkidou Alkistis. Papadopoulos Fotios C. Gender dysphoria in twins: a register-based population study. Scientific Reports. 2022. 12 (1). 10.1038/s41598-022-17749-0. PMC9352732 35927439

[CIT0010] Koch, H. L. (1966). *Twins and twin relations*. U. Chicago Press.

[CIT0011] Moilanen, I., Linna, S. L., Ebeling, H., Kumpulainen, K., Tamminen, T., Piha, J., & Almqvist, F, (1999). Are twins’ behavioural/emotional problems different from singletons. *European Child & Adolescent Psychiatry*, *8*(S4), S62–S67. 10.1007/PL00010702 10654135

[CIT0012] Neyer, F. J. (2002). Twin relationships in old age: A developmental perspective. *Journal of Social and Personal Relationships*, *19*(2), 155–177. 10.1177/0265407502192001

[CIT0013] Parker, E., & Davis-McCabe, C. (2021). The sibling experience: Growing up with a trans sibling. *Australian Journal of Psychology*, *73*(2), 188–199. 10.1080/00049530.2021.1882269

[CIT0014] Polderman, T. J. C., Kreukels, B. P. C., Irwig, M. S., Beach, L., Chan, Y. M., Derks, E. M., Esteva, I., Ehrenfeld, J., Heijer, M. D., Posthuma, D., Raynor, L., Tishelman, A., & Davis, L. K., International Gender Diversity Genomics Consortium (2018). The biological contributions to gender identity and gender diversity: bringing data to the table. *Behavior Genetics*, *48*(2), 95–108. 10.1007/s10519-018-9889-z 29460079

[CIT0015] Pulkkinen, L., Kokko, K., & Rantanen, J. (2012). Paths from socioemotional behavior in middle childhood to personality in middle adulthood. *Developmental Psychology*, *48*(5), 1283–1291. 10.1037/a0027463 22352969

[CIT0016] Riggs, D. W., Skelton, S., Katz-Wise, S. L., Sansfaçon, A. P., & Arora, M. (2025). Families with more than one trans person: Investments and divestments in cisnormativity. *Journal of Marriage and Family*, *87*(2), 812–823. 10.1111/jomf.13035

[CIT0017] Robin, M., & Casati, I. (1994). Are twins different from singletons during early childhood? *Early Development and Parenting*, *3*(4), 211–221. 10.1002/edp.2430030404

[CIT0018] Rutter, M., & Redshaw, J. (1991). Growing up as a twin: Twin-singleton differences in psychological development. *Journal of Child Psychology and Psychiatry, and Allied Disciplines*, *32*(6), 885–895. 10.1111/j.1469-7610.1991.tb01916.x 1744192

[CIT0019] Segal, N. L. (1999). *Entwined lives: Twins and what they tell us about human behavior.* Dutton/Penguin Books. 10808244

[CIT0020] Sofaer, S. (1999). Qualitative methods: What are they and why use them? *Health Services Research*, *34*(5 Pt 2), 1101–1118. PMC1089055 10591275

[CIT0021] Tancredy, C. M., & Fraley, R. C. (2006). The nature of adult twin relationships: An attachment-theoretical perspective. *Journal of Personality and Social Psychology*, *90*(1), 78–93. 10.1037/0022-3514.90.1.78 16448311

[CIT0022] Tenny, S., Brannan, J. M., & Brannan, G. D. (2023). *Qualitative Study*. 29262162

[CIT0023] Vears, D. F., & Gillam, L. (2022). Inductive content analysis: A guide for beginning qualitative researchers. *Focus on Health Professional Education: A Multi-Professional Journal*, *23*(1), 111–127. 10.11157/fohpe.v23i1.544

[CIT0024] Watzlawik, M. (2009). The perception of similarities and differences among adolescent siblings: Identification and deidentification of twins and nontwins. *Journal of Adolescent Research*, *24*(5), 561–578. 10.1177/0743558409338301

[CIT0025] Westwater, J. J., Riley, E., & Peterson, G. M. (2020). Using circular questions to explore individual family member experiences of youth gender dysphoria in Australia. *International Journal of Transgender Health*, *21*(3), 321–336. 10.1080/26895269.2020.1777616 PMC8726649 34993512

[CIT0026] Wheeler, N. L., Langton, T., Lidster, E., & Dallos, R. (2019). Understanding more about how young people make sense of their siblings changing gender identity: How this might affect their relationships with their gender-diverse siblings and their experiences. *Clinical Child Psychology and Psychiatry*, *24*(2), 258–276. 10.1177/1359104519830155 30968722

[CIT0027] Zaliznyak, M., Yuan, N., Bresee, C., Freedman, A., & Garcia, M. M. (2021). How early in life do transgender adults begin to experience gender dysphoria? Why this matters for patients, providers, and for our healthcare system. *Sexual Medicine*, *9*(6), e100448. 10.1016/j.esxm.2021.100448 PMC8766261 34731778

