# Supplementary material for: Gender diversity and the twin relationship: “If I’m feeling this way, shouldn’t my twin be feeling this way too?”
Source: Int J Transgend Health. 2025 Nov 7;27(5):2570–8. doi: 10.1080/26895269.2025.2552337 (PMC13618197; doi:10.1080/26895269.2025.2552337)
Supplement: Supplementary file 1 [file WIJT_A_2552337_SM7658.docx]

**SUPPLEMENTARY TABLES EXTENDED DATA**

**Supplementary data 1: Interview question guide (sample questions)**

The semi-structured nature of the interviews allowed for any relevant questions from this list to be asked. Additionally, any questions or topics that arose during the interview were also asked.

*Personal identity:*

1. Tell me about your gender journey.

2. Did you have a “coming out” experience or have you identified this way for as long as you can remember?

3. Can you tell me about your gender and how you identify?

*Twin relationship:*

1. Describe your relationship with your twin.

2. What are the biggest similarities and differences between you and your twin?

3. What were you and your twin like growing up?

4. Tell me about any times you have been extremely close or extremely distant with your twin.

5. How did other people perceive you and your twin growing up, as a pair or very separate?

6. Explain your experience of dependence with your twin.

7. Explain your experience(s) of comparing yourself to your twin.

8. Compare your relationship to your twin to your other siblings.

*Twin relation and trans experience:*

1. Tell me about your “coming out” experience to one another.

2. Tell me about the level of support from your twin in your gender journey.

3. Would you say having a twin helped or slowed down your coming out experience? How so?

4. How has the closeness between you and your twin changed after coming out?

5. Do you think whoever came out first made it easier or more difficult to come out?

6. Did you and your twin talk about your gender expression or dysphoria growing up?

7. Can you tell me about any conversations you had with your twin about gender identity.

8. Can you recall the emotions you experienced when your twin came out to you?
